# Bio-SCAN V2: A CRISPR/dCas9-based lateral flow assay for rapid detection of theophylline

**DOI:** 10.3389/fbioe.2023.1118684

**Published:** 2023-01-19

**Authors:** Wenjun Jiang, Rashid Aman, Zahir Ali, Magdy Mahfouz

**Affiliations:** Laboratory for Genome Engineering and Synthetic Biology, Division of Biological Sciences, King Abdullah University of Science and Technology (KAUST), Thuwal, Saudi Arabia

**Keywords:** CRISPR-dCas9, biotin labeling, lateral flow assay, non-nucleic acid detection, theophylline

## Abstract

Rapid, specific, and robust diagnostic strategies are needed to develop sensitive biosensors for small molecule detection, which could aid in controlling contamination and disease transmission. Recently, the target-induced collateral activity of Cas nucleases [clustered regularly interspaced short palindromic repeats (CRISPR)-associated nucleases] was exploited to develop high-throughput diagnostic modules for detecting nucleic acids and small molecules. Here, we have expanded the diagnostic ability of the CRISPR-Cas system by developing Bio-SCAN V2, a ligand-responsive CRISPR-Cas platform for detecting non-nucleic acid small molecule targets. The Bio-SCAN V2 consists of an engineered ligand-responsive sgRNA (ligRNA), biotinylated dead Cas9 (dCas9-biotin), 6-carboxyfluorescein (FAM)-labeled amplicons, and lateral flow assay (LFA) strips. LigRNA interacts with dCas9-biotin only in the presence of sgRNA-specific ligand molecules to make a ribonucleoprotein (RNP). Next, the ligand-induced ribonucleoprotein is exposed to FAM-labeled amplicons for binding, and the presence of the ligand (small molecule) is detected as a visual signal [(dCas9-biotin)-ligRNA-FAM labeled DNA-AuNP complex] at the test line of the lateral flow assay strip. With the Bio-SCAN V2 platform, we are able to detect the model molecule theophylline with a limit of detection (LOD) up to 2 μM in a short time, requiring only 15 min from sample application to visual readout. Taken together, Bio-SCAN V2 assay provides a rapid, specific, and ultrasensitive detection platform for theophylline.

## Introduction

Small-molecule detection is vital in drug discovery, metabolomics, and environmental monitoring. Most current analytical methods rely on sophisticated instruments and expensive reagents (Dutta et al., 2018; Amalfitano et al., 2021; Chang et al., 2021). Thus, rapid, economical, and user-friendly methods for small-molecule detection are highly sought after to increase the detection potential of the diagnostic toolbox. The discovery of clustered regularly interspaced short palindromic repeats (CRISPR) and CRISPR-associated (Cas) proteins have revolutionized various biological applications, including genome editing and medical diagnostics (Mahas et al., 2018; Aman et al., 2020; Zhang et al., 2022). The CRISPR-Cas-based nucleic acid detection system is simple, highly sensitive, and amenable for point-of-care diagnostics. The outbreak of Severe Acute Respiratory Syndrome Coronavirus 2 (SARS-CoV-2) highlighted the importance of point-of-care diagnostics and multiple CRISPR-Cas systems such as SHERLOCK, MORIARTY, and OPTIMA-dx were harnessed for rapid nucleic acid detection (Kellner et al., 2019; Sridhara et al., 2021; Mahas et al., 2022a).

While most of the reported CRISPR-Cas systems were developed for detecting nucleic acids, some studies reported using CRISPR-Cas systems for detecting small molecules (Liang et al., 2019; Iwasaki and Batey, 2020; Xiong et al., 2020). These studies represented advances in small molecule detection but also suffered from some drawbacks. For example, Liang et al. (2019) developed a CRISPR-Cas12a- and aTF-mediated small molecule detector (CaT-SMelor) system for small molecule detection, in which bacterial allosteric transcription factors were involved in the release of double-stranded DNA (dsDNA) in the presence of small molecule targets, resulting in the activation of Cas12a reporter cleavage activity. However, this system required multiple steps including washing, centrifugation, and facility-assisted readouts. We recently developed a Cas12a-based system for tetracycline detection where the *in vitro* transcription of CRISPR RNA (crRNA) followed by Cas12a binding and reporter cleavage was coupled to the presence of allosteric transcription factors (Mahas et al., 2022b). Though the platform allows the portable and sensitive detection of tetracycline, the reaction takes over an hour for readouts (Mahas et al., 2022b). Additionally, CRISPR-Cas-based detection systems have only been reported for limited types of molecules such as ATP, tetracycline, and glucose (Feng et al., 2021; Cheng et al., 2022; Samanta et al., 2022). Thus, there is a pressing need to explore more accessible and user-friendly strategies to expand the power of the CRISPR-Cas system for small molecule detection.

The CRISPR-Cas9 system, which uses Cas9 as the only effector and complementary RNA for DNA-ribonucleic protein (RNP) complex formation, is the most straightforward and widely used CRISPR system. Cas9 can form an RNP with crRNA and trans-activating crRNA (tracrRNA), later converted into a single-guide RNA (sgRNA). Guided by sequence complementarity, sgRNA-Cas9 binds and cleaves dsDNA in a sequence-dependent manner *via* its RuvC and HNH domains (Adli, 2018; Pickar-Oliver and Gersbach, 2019). However, specific binding of Cas9-sgRNA is independent of double-stranded DNA cleavage. Cas9 nickase (nCas9) and nuclease-dead Cas9 (dCas9) can be generated by mutating one or both of its endonuclease domains, respectively (Kleinjan et al., 2017; Butt et al., 2020; Volke et al., 2022). Many strategies have also been developed for regulating CRISPR-Cas9 activities (Polstein and Gersbach, 2015; Liu et al., 2016; Lundin et al., 2020). Kale et al. described the ligand-responsive CRISPR-dCas9 system by inserting aptamers into sgRNA to obtain ligand-responsive sgRNA (ligRNA). In the presence of ligands, ligRNA becomes activated, resulting in CRISPR-Cas9-based gene repression in bacterial systems (Kundert et al., 2019). By incorporating the ligand-responsive self-cleaving aptazyme into guide RNAs, Tang et al. (2017) also developed small-molecule-controlled CRISPR-Cas9-mediated genome editing and a transcriptional regulation platform in mammalian cells. Though ligand-responsive sgRNA has been employed to regulate CRISPR-Cas9 activities in cells, no studies have reported its application in small molecule diagnostics to date.

Benefiting from their rapid, economical, and user-friendly properties, lateral flow assays (LFAs) have become one of the most reliable tools for medical diagnostics (Wang et al., 2020; Barnes et al., 2020; Arizti-Sanz et al., 2022). Cas9 has recently been coupled with LFA for antigen detection. The visual output of Cas9-mediated LFA detection relies on the incorporation of 6-carboxyfluorescein (FAM) and biotin moieties into the detection complex (Wang et al., 2020; Xiong et al., 2021). However, these systems require other components, such as an AuNP-DNA (gold, aurum nanoparticle-DNA) probe, customized LFA strips, and unconventional reporters, which are not feasible with point-of-care and low-resource diagnostic settings. To simplify the CRISPR-Cas9-LFA-mediated detection assay, we previously developed a biotin-labeled dCas9 (Bio-dCas9) system called Bio-SCAN for LFA-based nucleic acid detection. Bio-SCAN facilitated rapid and sensitive detection of SARS-CoV-2 and showed great potential for other applications in diagnostics (Ali et al., 2022).

To enable dCas9-biotin detection of various non-nucleic acid molecules, we coupled dCas9-biotin with a ligand-responsive sgRNA to generate a small molecule detection system called Bio-SCAN V2. For Bio-SCAN V2, dCas9-biotin was produced and purified from *Escherichia coli*. LigRNA was obtained by inserting a ligand-responsive aptamer into sgRNA. FAM-labeled amplicons were prepared by PCR amplification of the desired DNA sequence with FAM-labeled primers. Engineering of sgRNA (ligRNA, insertion of aptamer sequence) disabled ligRNA-(dCas9-biotin) RNP formation, thus blocking its binding to DNA. In the presence of the ligand, the ligRNA-(dCas9-biotin) interaction is restored, allowing (dCas9-biotin)-ligRNA-FAM labeled DNA assembly. Next, the application of (dCas9-biotin)-ligRNA-FAM labeled DNA onto the LFA strip immobilized the gold nanoparticle-(anti-FAM) antibody (αFAM antibody-AuNP) at the test line for visual detection. To demonstrate the practical application of Bio-SCAN V2, we engineered sgRNAs with a specific aptamer sequence responsive to theophylline. As one of the most commonly used anti-asthmatic drugs, theophylline has a narrow therapeutic index (20 µM–100 µM). Therefore, it is important to develop novel methods to facilitate theophylline concentration monitor (Jiang et al., 2015). Our experimental results demonstrated that the Bio-SCAN V2 platform can detect theophylline with high sensitivity and specificity in a 15-min sample-to-results readout time. We envision that with ligand-specific designs, the Bio-SCAN V2 assay can be reprogrammed for the detection of a wide range of small molecules in resource-limited settings.

## Materials and methods

### Protein purification

Biotin-labeled dCas9 (Bio-dCas9) preparation and validation of biotin-labeling was performed following our previous protocol (Ali et al., 2022). Briefly, the coding sequence of AviTag was cloned in-frame and downstream of the *dCas9* sequence, along with the *BirA* gene for biotinylation of the AviTag, into the pET28a plasmid to generate pET28a-dCas9-AviTag-BirA. This plasmid was then transformed into *E. coli* strain BL21 (*DE3*) cells for protein expression. Cells were grown in 2x YT medium with 50 mg/L kanamycin sulfate at 37°C. After reaching an OD600 of 0.7 mM, 0.5 mM isopropyl β-D-thiogalactopyranoside (IPTG) for protein induction and 100 μM biotin for biotinylation of dCas9-AviTag were added. The bacterial cultures were further incubated for 16 h at 18°C. The bacteria were collected by centrifugation at 6,000 *g* for 20 min and then lysed in lysis buffer (2 mg/mL lysozyme, 50 mM Tris−HCl pH 8.0, 300 mM NaCl, 20 mM imidazole, 0.1% [v/v] NP-40, 1 mM PMSF, 5% [v/v] glycerol, and EDTA-free protease inhibitor cocktail tablet/50 mL [Roche, United Kingdom]). The lysate was purified by an AKTA Pure system with a HisTrap HP 5 mL affinity column (GE Healthcare) and then with a HiLoad Superdex 16/600,200 pg gel filtration column (GE Healthcare). The protein concentration was measured using a spectrophotometer (Thermo Scientific NanoDrop 8,000) and the protein was collected, concentrated, flash-frozen, and stored at −80°C.

### *In vitro* transcription of ligRNAs and preparation of dCas9-biotin target

LigRNA coding sequences were obtained as a sense strand appended with an upstream T7 promoter and an antisense strand (Supplementary Table S1). A complementary region was designed for annealing between the sense and antisense strands (ligRNA1-1 and ligRNA1-2 for ligRNA1, ligRNA2-1 and ligRNA2-2 for ligRNA2, ligRNA3-1 and ligRNA3-2 for ligRNA3, ligRNA4-1 and ligRNA4-2 for ligRNA4). The two oligonucleotides were annealed in 1X PCR buffer (−MgCl_2_; Invitrogen), starting with denaturation at 95°C for 5 min, followed by 5°C ramp downsteps to 4°C. The annealed products were then amplified by PCR (with primer T7-F and individual ligRNA-R primers for ligRNA1–4) and purified (QIAquick PCR Purification Kit, QIAGEN). 1 µg of purified PCR amplicons was used as a template for *in vitro* transcription using Transcript Aid T7 High Yield Transcription Kit (Thermo Scientific, K0441) overnight at 37°C. The *in vitro* transcript was then purified with a Direct-zol RNA miniprep kit (R2050, Zymo Research). The concentration of purified ligRNAs was measured using a Nanodrop spectrophotometer (Thermo Scientific) and diluted into 6 µM working stocks. FAM-labeled amplicon for Bio-SCAN V2 execution was amplified with Target-F-FAM and Target-R primers using a TheoPCR-template as a template (Supplementary Table S2). The concentration of the 152-bp FAM-labeled amplicon was measured using a Nanodrop spectrophotometer (Thermo Scientific) and diluted into 300 ng/μL working stocks. The 370-bp target for the *in vitro* Cas9 cleavage experiment was amplified with Target-F-2 and Target-R primers using TheoPCR-template as a template (Supplementary Table S2). The amplified target concentration was measured using a Nanodrop spectrophotometer (Thermo Scientific) and diluted into 300 ng/μL working stocks.

### Validation of ligand-responsive sgRNA by *in vitro* Cas9 cleavage of target DNA

For the theophylline-responsive *in vitro* Cas9 cleavage experiment, 300 ng of the 370-bp target amplicon, Cas9 (final concentration 50 nM), ligRNAs (final concentration 50 nM) were combined in 1X buffer 3 (20 mM HEPES pH 7.5, 150 mM KCl, 10 mM MgCl_2_, 0.5 mM DTT) and incubated at 37°C for 10 min with or without 50 μM theophylline (Sigma-Aldrich, T1633-50G). After incubation, the samples were separated on 2% agarose gels and imaged using the FluorChem Q imaging System (ProteinSimple).

### Ligand detection *via* Bio-SCAN V2

For ligand detection, 10 µL of theophylline (final concentration 50 µM) was incubated with 50 nM dCas9-biotin, 50 nM ligRNA1 or ligRNA2 in a 20 μL 1X buffer 1 (NEBuffer 2.1, New England Biolabs) at room temperature (RT) for 5 min to allow RNP assembly. The RNP solution (the mixture of dCas9-biotin, ligRNA1 or ligRNA2, and buffer 1 as indicated above) without theophylline was treated as the negative control. Subsequently, 1 μL FAM-labeled amplicon (300 ng) and 79 μL HybriDetect buffer (Milenia Biotec) were added to 20 μL of RNP solution to bring the final reaction volume to 100 μL. After centrifugation and vortexing, the reaction mixture was incubated at 37°C for 10 min. HybriDetect Dipsticks (Milenia Biotec) were equilibrated to room temperature (RT), placed into each tube, and removed from tubes upon the appearance of control lines. The results were obtained within 5 minutes. The appearance of both test lines and control lines represented positive samples. The appearance of only test or control lines represented invalid and negative samples, respectively.

### Optimization of Bio-SCAN V2

To evaluate the effects of RNP concentration on the performance of Bio-SCAN V2, we used Bio-SCAN V2 for theophylline detection with various concentrations of the dCas9-biotin RNP. 10 µL theophylline (50 µM final concentration) was incubated with 10 nM, 30 nM, 50 nM, 75 nM, and 100 nM dCas9-biotin/ligRNA1 in 20 µL 1X buffer one solution at RT for 5 min. RNP solution without theophylline was treated as the negative control. The following steps are the same as ligand detection *via* Bio-SCAN V2. To test the effects of working temperature, 10 µL theophylline (50 µM final concentration) was incubated with 30 nM dCas9-biotin/ligRNA1 in 20 µL 1X buffer 1 at RT for 5 minutes. The RNP solution without theophylline was treated as the negative control. Subsequently, 1 µL FAM-labeled amplicon (300 ng) and 79 μL HybriDetect buffer was added to 20 μL RNP solution to bring the final reaction volume to 100 μL. After centrifugation and vortexing, the reaction mixture was incubated at RT, 37°C, 40°C, 45°C, or 50°C for 10 min. The following steps are the same as ligand detection *via* Bio-SCAN V2. To evaluate Bio-SCAN V2 function with various buffers, 10 µL theophylline (50 µM final concentration) was incubated with 30 nM dCas9-biotin/ligRNA1 in either 20 µL 1X buffer 1, 20 µL 1X buffer 2 (NEBbuffer 3.1) or 20 µL 1X buffer 3 at RT for 5 min. The RNP solution without theophylline was treated as the negative control. Bio-SCAN V2 was then executed at 45°C, following the same steps as ligand detection *via* Bio-SCAN V2.

### The specificity and robustness of Bio-SCAN V2

To evaluate the specificity of Bio-SCAN V2, we performed Bio-SCAN V2 for the detection of caffeine (Sigma-Aldrich, C0750-5G) and xanthine (Sigma-Aldrich, X0626-5G) together with theophylline. 10 µL theophylline, caffeine, or xanthine (50 µM final concentration) was incubated with 30 nM dCas9-biotin/ligRNA1 in 20 µL 1X buffer 3 solution at RT for 5 min. RNP solution without theophylline was treated as the negative control. The following steps are the same as ligand detection *via* Bio-SCAN V2. To check the robustness of Bio-SCAN V2 for testing, we performed Bio-SCAN V2 for the detection of theophylline with the supplements of caffeine and Bovine serum albumin (BSA) as interferents. 10 µL theophylline (50 µM final concentration) with the supplement of 10 mM BSA and 50 µM caffeine was incubated with 30 nM dCas9-biotin/ligRNA1 in 20 µL 1X buffer 3 solution at RT for 5 min. RNP solution without theophylline was treated as the negative control. The following steps are the same as ligand detection *via* Bio-SCAN V2.

### Limit of detection (LOD) for Bio-SCAN V2

Theophylline (final concentrations of 0 µM, 1 µM, 2 µM, 5 µM, 10 µM, 30 µM, 100 µM, 300 µM, 1,000 µM, and 2,000 µM) was incubated with 30 nM dCas9-biotin and 30 nM ligRNA1 in 20 µL 1X buffer 3 solution at RT for 5 min. The 20 µL solutions of RNP were then incubated with 1 μL FAM-labeled amplicon (300 ng) and 79 μL HybriDetect buffer at 45°C for 5 min. HybriDetect Dipsticks were equilibrated to RT and immediately placed into each tube. The dipsticks were removed from tubes as soon as the control lines appeared. To determine theophylline concentration, linear regression between relative band intensity and the log value of theophylline concentration was calculated by GraphPad Prism 9.

### Image analysis of lateral-flow reactions

The relative intensity of lateral-flow strips was analyzed by ImageJ as described by Kaminski et al. (2020). Images of lateral-flow strips were first converted to grayscale and inverted. Relative band intensity was calculated by the mean gray value of the test band divided by that of the control band.

### Data processing and visualization

All raw data were analyzed and visualized by GraphPad Prism 9. All numerical data were presented as mean ± standard deviation (SD). Two-tailed Student *t*-tests were selected for statistical analysis to detect differences between the negative control and experimental groups.

## Results

### Design and construction of Bio-SCAN V2

**FIGURE 1:**
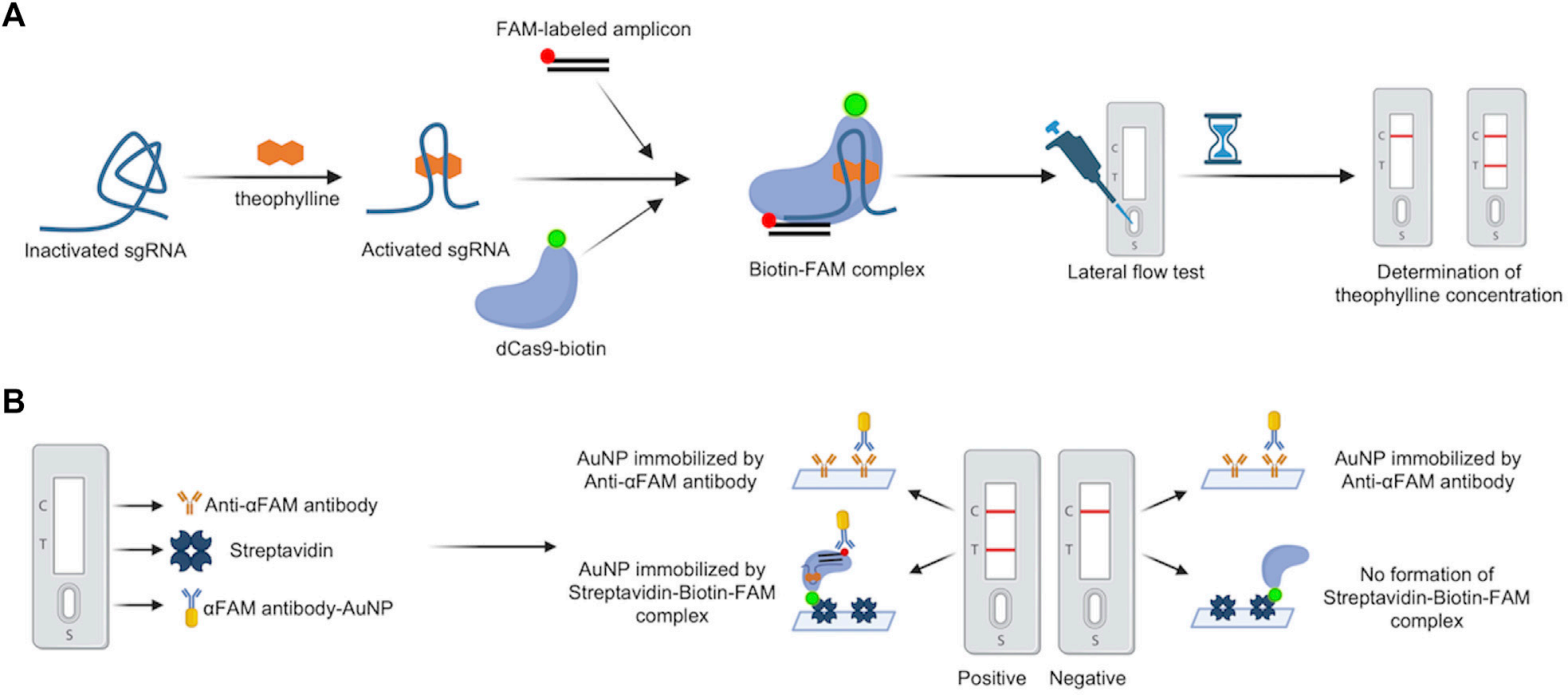
Schematic diagram of the Bio-SCAN V2. **(A)** The principle of Bio-SCAN V2-based theophylline detection. ligRNA remains inactivated without theophylline. With the presence of theophylline, ligRNA would be re-activated to form the dCas9-biotin-ligRNA-FAM-DNA complex with dCas9-biotin and FAM-labeled amplicon. The streptavidin-coated test line captures the dCas9-biotin-ligRNA-FAM-DNA-AuNP complex, which is displayed as a visual signal readout on the LFA strip. The concentration of theophylline can be determined by quantitative analysis of the relative test line intensity. Control line (C), Test line (T). **(B)** Composition of the LFA strip used in the study. Sample pad (S) contains αFAM antibody-AuNP; Test line (T) is modified by Streptavidin; Control line (C) is covered by Anti-αFAM antibody. The αFAM antibody-AuNP will be immobilized by the Streptavidin-Biotin-FAM complex to generate bands at the test line in positive samples. The excessive αFAM antibody-AuNP will be immobilized by the Anti-αFAM antibody to generate bands at the control line in both positive and negative samples.

Bio-SCAN V2 was engineered from our previously reported Bio-SCAN system (Ali et al., 2022). In Bio-SCAN, we developed the biotinylated dead Cas9 (dCas9-biotin) for nucleic acid detection. During the lateral flow assay (LFA), dCas9-biotin RNP can detect a FAM-labeled amplicon, which is only produced in the presence of the nucleic acid target. For Bio-SCAN V2, we sought to design ligand-responsive sgRNA (ligRNA), which could be activated by small non-nucleic acid target molecules. The presence of target small molecules in the form of activated ligRNA is coupled with the dCas9-biotin/FAM-amplicon/αFAM antibody-AuNP complex on commercially available LFA strips for visual signal readout. To validate our CRISPR-Cas9-based small molecule detection strategy, we engineered a molecular platform called Bio-SCAN V2 for theophylline detection (Figure 1). The Bio-SCAN V2 assay requires a FAM-labeled amplicon, dCas9-biotin, ligRNA, LFA strips, and the target ligand molecule for assay actuation. FAM-labeled nucleic acids and dCas9-biotin were produced following our previous protocol (Ali et al., 2022). For ligand-induced sgRNAs, we engineered multiple ligRNAs and selected one reported sgRNA (ligRNA1) activated by theophylline, as previously shown (Kundert et al., 2019). All three ligRNAs (ligRNA2, ligRNA3, ligRNA4) were engineered by inserting the theophylline aptamer reported by Bayer et al. (Bayer and Smolke, 2005) into the hairpin structure of standard sgRNA (for Cas9) *via* a strand-displacement strategy. The sequences of all four ligRNAs are listed in Supplementary Table S3. The Bio-SCAN V2 assay was actuated by assembling (dCas9-biotin)-ligRNA-FAM labeled DNA in the presence and absence of target small molecules and application of the RNP-DNA complex to the αFAM antibody-AuNP embedded part of the LFA strips. The presence of the target molecule gives rise to the activation of (dCas9-biotin)-ligRNA-FAM labeled DNA complex formation and immobilization of the established (dCas9-biotin)-ligRNA-FAM labeled DNA-AuNP complex at the test line. As a result, the visual signal band is observed on the LFA strip. Unbound αFAM antibody-AuNP is detected at the control line. In the absence of target small molecules, no RNP and further (dCas9-biotin)-ligRNA-FAM labeled DNA-AuNP complex formation results in no visual band at the test line on the LFA strip.

### The Bio-SCAN V2 is responsive to theophylline

**FIGURE 2:**
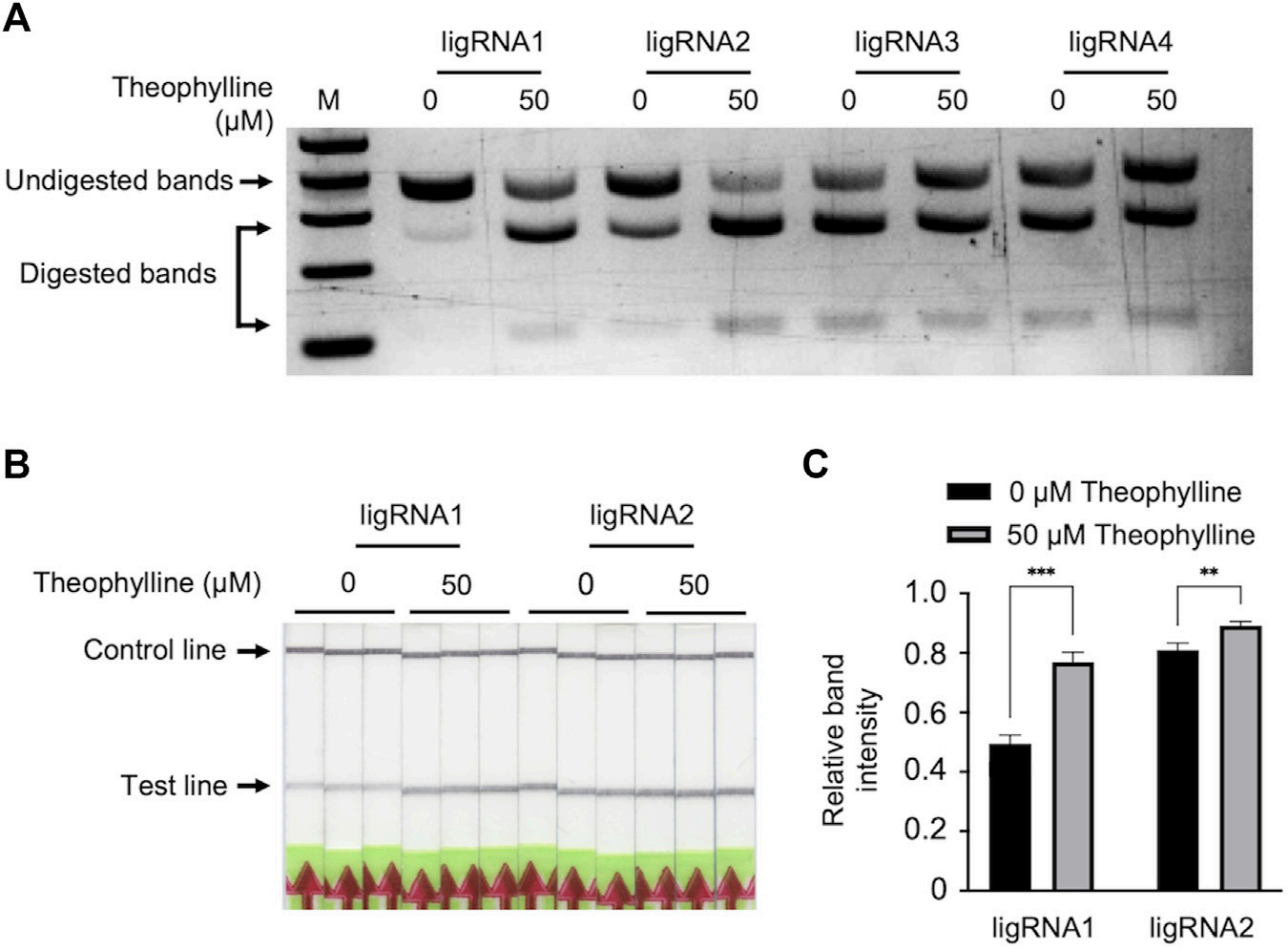
Detection of theophylline by Bio-SCAN V2. **(A)** Theophylline-responsive *in vitro* Cas9 cleavage assay with different ligRNAs (1–4). To validate the theophylline-responsive properties of the ligRNAs, a 370-bp target was digested by Cas9 guided by ligRNA1, ligRNA2, ligRNA3, and ligRNA4 individually (left to right) with 50 μM theophylline or without theophylline. Arrowheads indicate the digested DNA. **(B)** LigRNA1-and ligRNA2-mediated lateral flow assay for theophylline detection. Black arrows indicate the expected locations of the test line and control line. **(C)** Quantitative analysis of relative test line intensity of Bio-SCAN V2-based theophylline detection using ImageJ software. Data are expressed as mean ± standard deviation (SD) (*n* = 3).

To evaluate the ligand-induced activity of our engineered ligRNAs, we first performed an *in vitro* cleavage assay of a 370-bp PCR-amplified product using Cas9-ligRNAs in the presence and absence of theophylline. The PCR-amplified target was incubated with catalytically active Cas9 and four ligRNAs individually, in the presence or absence of 50 μM theophylline at 37°C for 10 min. The cleavage products were separated on a 2% agarose gel. As shown in Figure 2A, ligRNA1 and ligRNA2 showed clear Cas9-mediated DNA cleavage activity in the presence of 50 μM theophylline compared to without theophylline. Moreover, ligRNA1 demonstrated specific, high Cas9 cleavage activity in the presence of 50 μM theophylline compared to ligRNA2. The cleavage patterns of Cas9 mediated by ligRNA3 and ligRNA4 did not greatly differ in the presence or absence of 50 μM theophylline. Our results confirmed that ligRNA1 and ligRNA2 have good theophylline-responsive properties, which can potentially be developed for theophylline detection. Therefore, we selected ligRNA1 and ligRNA2 to develop the Bio-SCAN V2 system.

We next tested the Bio-SCAN V2 for theophylline detection with ligRNA1 and ligRNA2. To accomplish this, ligRNA1 or ligRNA2, dCas9-biotin, and FAM-labeled amplicon were combined and incubated with or without 50 μM theophylline at 37°C for 10 min. The reaction mixture was applied to LFA strips for visual detection without further dilution. As shown in Figure 2B, test lines were observed when executing Bio-SCAN V2 without theophylline. However, the ligRNA1 groups showed stronger test lines in the presence of 50 μM theophylline when compared to the respective negative control. On the other hand, clear bands were observed for ligRNA2 when running the Bio-SCAN V2 reaction both with and without 50 μM theophylline. Relative band intensity was quantified using ImageJ. As shown in Figure 2C, statistically significant differences were observed between ligRNA1 and ligRNA2 groups with 50 μM theophylline and without theophylline. Our results confirmed that Bio-SCAN V2 can potentially be harnessed as a tool for rapid *in vitro* detection of theophylline. We selected ligRNA1 for subsequent experiments, as it demonstrated better theophylline-responsive properties. However, there was still strong background observed in the ligRNA1 LFA results without theophylline. Thus, further optimization is required to adopt Bio-SCAN V2 for theophylline detection.

### Bio-SCAN V2 successfully detected theophylline

**FIGURE 3:**
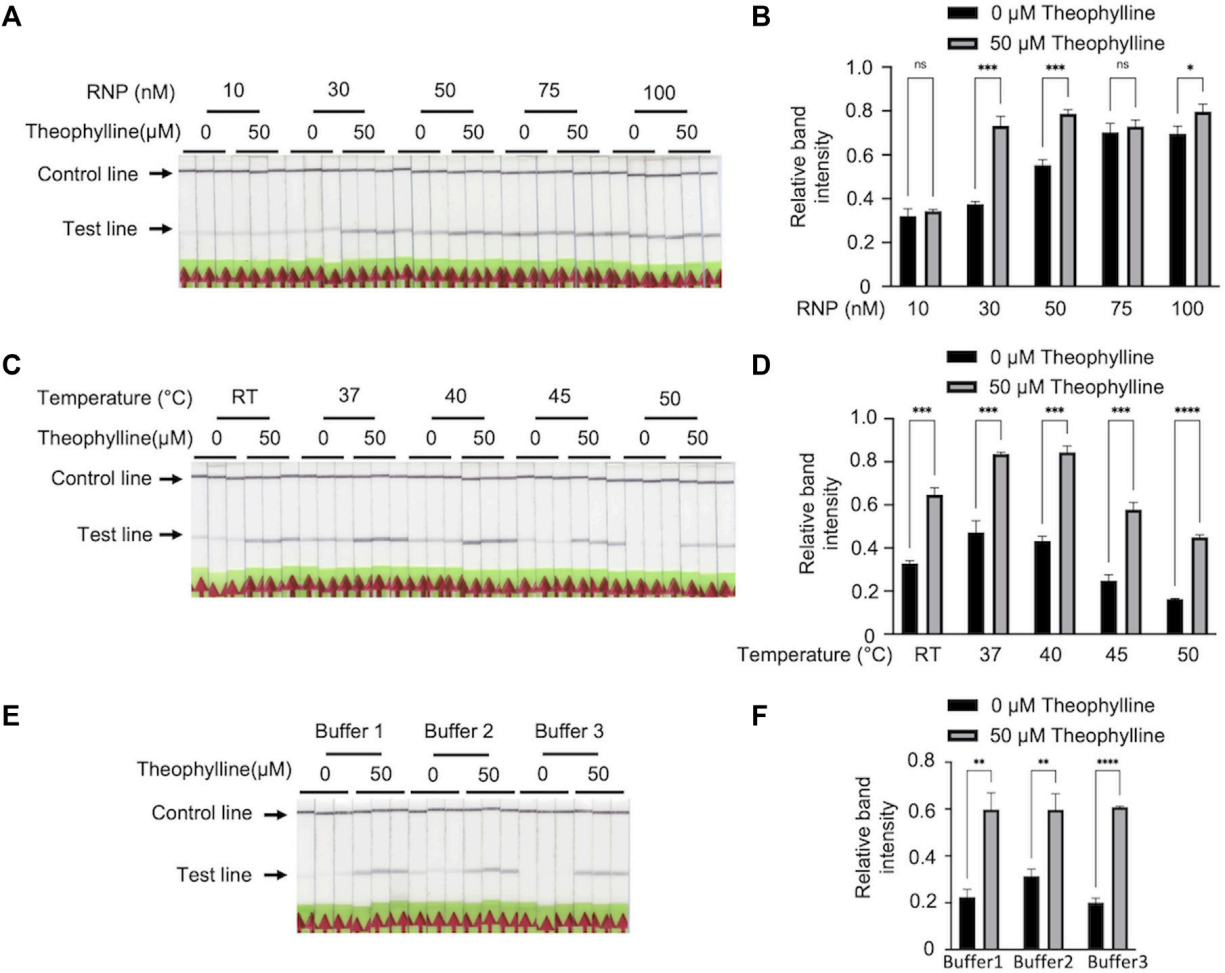
Optimization of Bio-SCAN V2 for theophylline detection. **(A)** Optimization for RNP complex concentration. A Bio-SCAN V2 assay was performed to detect theophylline using varying concentrations of RNP complex (10 μM, 30 μM, 50 μM, 75 μM, and 100 μM). **(B)** Quantification of relative test line intensity of the Bio-SCAN V2 results obtained in **(A)** using ImageJ software. **(C)** Optimization of detection assay temperature. Bio-SCAN V2 assays were assembled at room temperature, 37°C, 40°C, 45°C, and 50°C using 0 μM and 50 μM theophylline. **(D)** Quantification of relative test line intensity of the Bio-SCAN V2 results obtained in **(C)** using ImageJ software. **(E)** Selection of efficient buffer composition for Bio-SCAN V2 assays. Bio-SCAN V2 assays were performed with buffer 1, buffer 2, and buffer 3 to determine a compatible buffer for detection of theophylline. **(F)** Quantification of relative test line intensity of the Bio-SCAN V2 results obtained in **(E)** using ImageJ software. Data are expressed as mean ± SD (*n* = 3). The black arrowheads indicate the expected locations of the test line and control line.

To enhance Bio-SCAN V2 specificity, we evaluated several parameters to achieve robust theophylline detection without background on LFA strips. First, we investigated the effect of RNP concentration on Bio-SCAN V2 performance. We carried out theophylline detection assays using different concentrations of ligRNA-(dCas9-biotin) RNP complex (10 nM, 30 nM, 50 nM, 75 nM, and 100 nM). The LFA results demonstrated that with 30 nM dCas9-biotin RNP, the Bio-SCAN V2 efficiently detected 50 μM theophylline on LFA strips, compared to no detection with 10 nM and high background in the case of 50 nM, 75 nM, 100 nM dCas9-biotin RNP (Figure 3A). The quantitative analysis clearly determined that the Bio-SCAN V2 performed best with an RNP concentration of 30 nM to detect 50 μM theophylline (Figure 3B). We next evaluated the effect of temperature on the Bio-SCAN V2 using the optimal 30 nM dCas9-biotin RNP. A Bio-SCAN V2 assay for theophylline detection was performed at room temperature, 37°C, 40°C, 45°C, and 50°C. LFA results demonstrated that Bio-SCAN V2 detected theophylline efficiently at 45°C and 50°C with lower background band appearance compared to room temperature, 37°C, and 40°C (Figures 3C, D). A working temperature of 45°C was selected for theophylline detection *via* Bio-SCAN V2. Next, we optimized the buffer requirements of Bio-SCAN V2. As shown in Figures 3E, F, Bio-SCAN V2 detected theophylline in buffer 3 without any background, compared to buffers 1 and 2. These results confirmed that through optimization of dCas9-biotin RNP concentration, temperature, and buffer, Bio-SCAN V2 can be efficiently adapted for the detection of small molecules including theophylline.

### The specificity and robustness of Bio-SCAN V2

**FIGURE 4:**
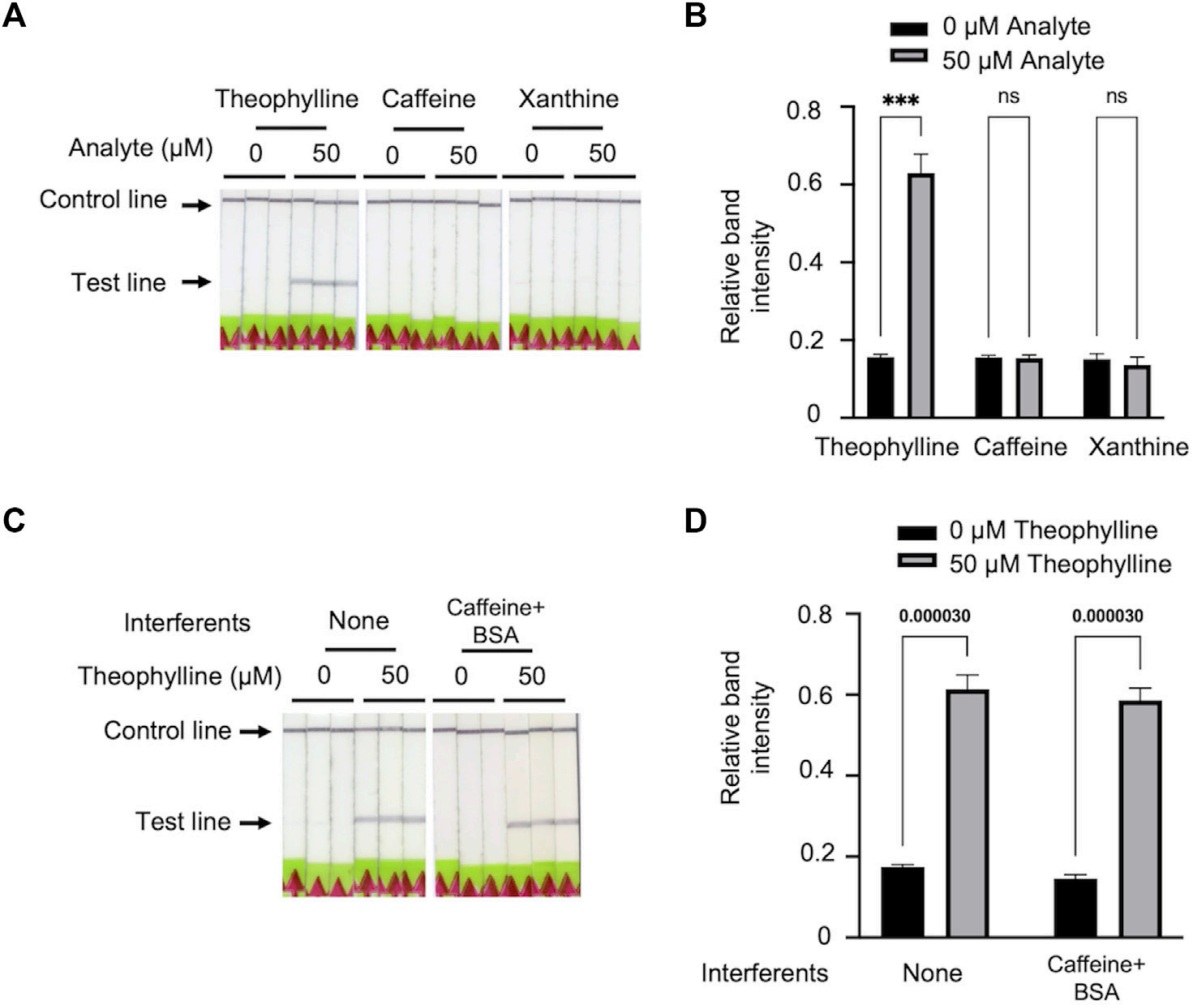
The specificity and robustness of Bio-SCAN V2 for theophylline detection. **(A)** Bio-SCAN V2 was performed to detect 50 μM theophylline, 50 μM caffeine, and 50 μM xanthine for the validation of specificity. **(B)** Quantitative analysis of relative test line intensity of Bio-SCAN V2-based xanthine derivates detection using ImageJ software. Data are expressed as mean ± SD (*n* = 3). **(C)** Bio-SCAN V2 was performed to detect 50 μM theophylline with interferents for the robustness test. **(D)** Quantitative analysis of relative test line intensity of Bio-SCAN V2-based theophylline detection with interferents using ImageJ software. Data are expressed as mean ± SD (*n* = 3), and *p*-values are shown for two-tailed Student *t*-tests.

We selected xanthine and caffeine as the analogues to validate the specificity of Bio-SCAN V2. Among all three analytes, Bio-SCAN V2 was only able to detect theophylline. No bands were observed with samples supplemented with 50 μM xanthine or 50 μM caffeine (Figures 4A, B). Our results demonstrate that the Bio-SCAN V2 has good specificity for theophylline detection. To explore the robustness of the system, theophylline samples for Bio-SCAN V2 were supplemented with 10 mM BSA and 50 μM caffeine to simulate the co-existence of analogues in protein-abundant environments. Compared with theophylline samples without interferents, the addition of BSA and caffeine did not influence the Bio-SCAN V2-based theophylline detection, suggesting the good robustness of Bio-SCAN V2 (Figures 4C, D).

### Limit of detection (LOD) of the optimized Bio-SCAN V2

**FIGURE 5:**
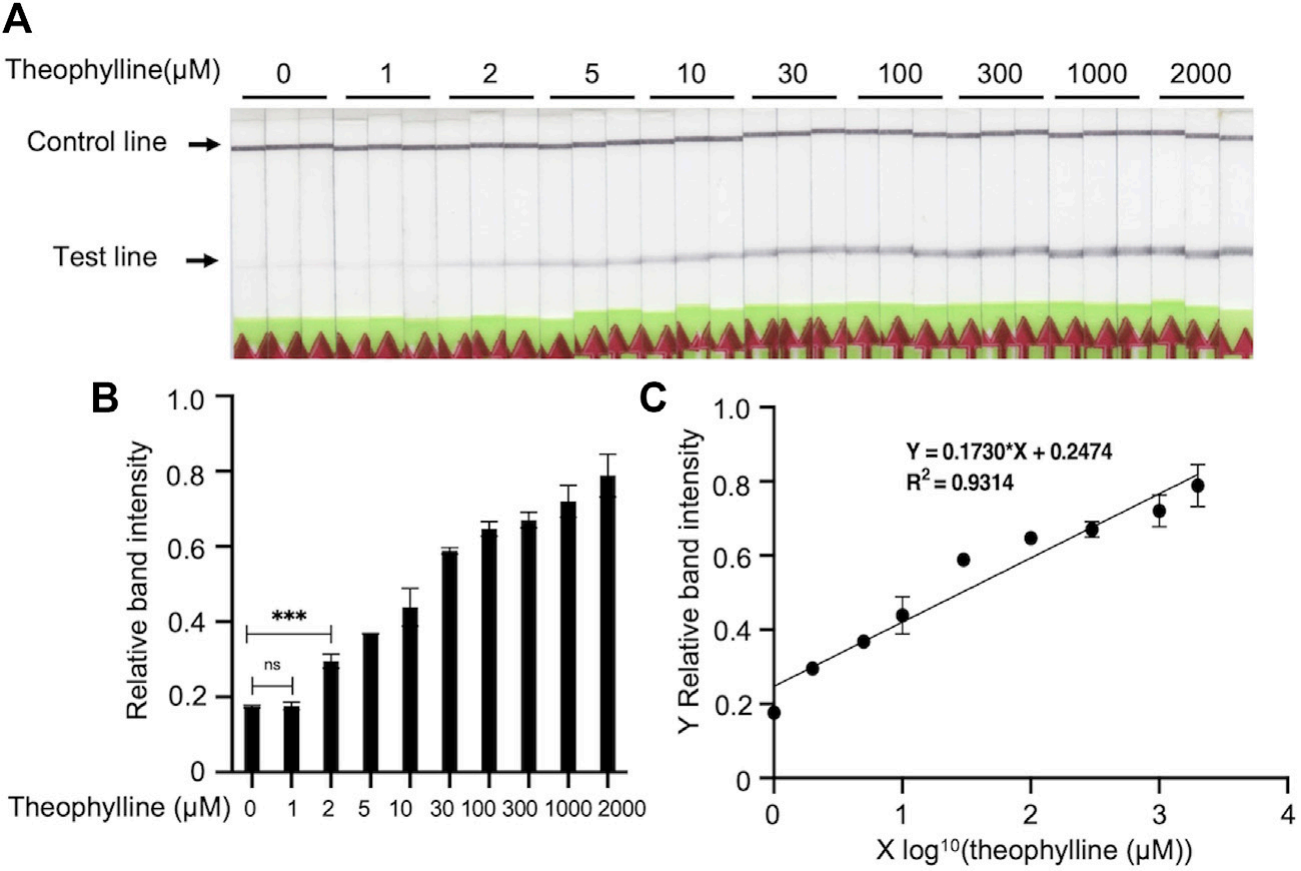
Limit of detection (LOD) determination of Bio-SCAN V2-based theophylline detection. **(A)** A Bio-SCAN V2 assay was performed to detect theophylline (0 μM, 1 μM, 2 μM, 5 μM, 10 μM, 30 μM, 100 μM, 300 μM, 1,000 μM, 2,000 μM) under optimized conditions. **(B)** Quantitative analysis of relative test line intensity of Bio-SCAN V2-based theophylline detection under the optimized conditions using ImageJ software. Data are expressed as mean ± SD (*n* = 3). The LOD was defined as the lowest concentration that significantly differs from groups with 0 μM theophylline. **(C)** Linear regression between the log value of theophylline concentration and relative band intensity determined using GraphPad Prism 9.

To determine the LOD of Bio-SCAN V2 for theophylline, a diagnostic assay was performed at optimized conditions to detect 0 μM, 1 μM, 2 μM, 5 μM, 10 μM, 30 μM, 100 μM, 300 μM, 1,000, and 2,000 μM theophylline. The LOD was defined as the lowest concentration showing a significant difference compared with the 0 μM theophylline groups. Our results revealed that Bio-SCAN V2 can detect as low as 2 μM theophylline (Figures 5A, B). Figure 5C illustrates the linear relationship between the log value of theophylline concentration and relative LFA band intensity. The concentration of theophylline can be calculated based on Y (relative LFA band intensity) = 0.1730*X (log value of theophylline concentration) + 0.2474.

## Discussion

We previously developed Bio-SCAN platform for the accurate detection of nucleic acids, requiring no sophisticated equipment or technical expertise (Ali et al., 2022). In this study, we reprogrammed Bio-SCAN for simple and fast detection of small molecules. To achieve this, we replaced the standard sgRNA in Bio-SCAN with a ligRNA (ligand-responsive) that gets activated in the presence of a specific small molecule. LigRNA can be generated by incorporating an aptamer or aptazyme into the sgRNA, which has been reported for ligRNA-responsive CRISPR/Cas9-based genome engineering in cells (Tang et al., 2017; Kundert et al., 2019). By coupling ligRNA with Bio-SCAN, we created Bio-SCAN V2, a CRISPR-Cas-based system for the detection of theophylline using theophylline-responsive ligRNA. The specificity and sensitivity of Bio-SCAN V2 depend on the theophylline that actuates the (dCas9-biotin)-ligRNA-FAM labeled DNA complex for signal readout on LFA strips. Among four tested ligRNAs, ligRNA1 showed high specificity for theophylline detection. The other ligRNAs, especially ligRNA 3 and ligRNA 4 showed poor theophylline specificity. One potential explanation is that the insertion of aptamer failed to disrupt the functional structure of sgRNA without theophylline in ligRNA3 and ligRNA 4 (Kundert et al., 2019). Furthermore, the theophylline-responsive properties of ligRNAs differed under different temperatures and buffer conditions. Therefore, we optimized dCas9-biotin RNP concentration, the working buffer, and temperature to enhance the performance of Bio-SCAN V2. As a result, Bio-SCAN V2 can be utilized to detect theophylline with LOD of 2 μM *via* LFA in 15 min, and the theophylline concentration can be further determined by quantitative analysis of relative band intensity.

High-performance liquid chromatography (HPLC) is the most commonly used method for theophylline detection (Srdjenovic et al., 2008; Al-Jenoobi et al., 2015). Though with high sensitivity and accuracy, HPLC analysis involves expensive costs on solvents, maintenance, and consumables such as columns. Therefore, novel economic methods for theophylline detection are required. As substitutes, different biosensors have been reported to detect theophylline, but each has its limitations (Jiang et al., 2015; Feng et al., 2018; Sett et al., 2021; Harding et al., 2022). For instance, Feng et al. (2018) proposed an aptamer-based nanopore film sensor for theophylline detection, which can detect theophylline with a LOD of 0.05 μM. However, the fabrication of the nanopore sensor and the surface functionalization increase platform complexity. Additionally, the system’s output relies on spectrometers, making it ill-suited for limited-resource settings. Harding et al. (2022) developed a fluorescent theophylline detection system by complexing deoxyribozymes with RNA aptamers. The system can provide an economical platform for theophylline detection but requires a reaction time as long as four hours. Chavez et al. (2010) reported a gold nanoparticle aggregation-based colorimetric biosensor, enabling portable visual detection of theophylline. However, the system can only detect theophylline in the range of 50 μM–240 μM. Compared with current theophylline detection platforms, Bio-SCAN V2 provides a good alternative for rapid and sensitive theophylline detection in resource-limited settings.

Similar to other detection platforms, Bio-SCAN V2 also possesses some limitations. Bio-SCAN V2 currently requires a working temperature of 40°C to eliminate background in the control groups. It would be beneficial if Bio-SCAN V2 can be performed with a clean background in control groups under RT. Though we tested the robustness of Bio-SCAN V2 with BSA and caffeine, the performance of Bio-SCAN V2 might be influenced by other interferents. It is highly recommended to re-obtain the standard quantitative curve with specific samples for practical applications.

Overall, we believe that the Bio-SCAN V2 system broadens the power of the CRISPR-Cas system for non-nucleic acid small molecule detection and presents a valuable advance to the small molecule detection toolbox.

## Data Availability

The raw data supporting the conclusion of this article will be made available by the authors, without undue reservation.

## Abbreviations

LFA, Lateral flow assay; dCas9-biotin, biotinylated dead Cas9; ligRNA, ligand-responsive Cas9 sgRNA; AuNP, gold nanoparticles; Bio-SCAN, biotin-coupled specific CRISPR-based assay for nucleic acid detection; LOD, limit of detection; CRISPR, clustered regularly interspaced short palindromic repeats; αFAM, anti-(6-carboxyfluorescein).
