# Supplementary material for: Bio-SCAN V2: A CRISPR/dCas9-based lateral flow assay for rapid detection of theophylline
Source: Front Bioeng Biotechnol. 2023 Jan 19;11:1118684. doi: 10.3389/fbioe.2023.1118684 (PMC9893010; doi:10.3389/fbioe.2023.1118684)
Supplement: Supplementary file 1 [file DataSheet1.PDF]

# Supplementary Information

**Bio-SCAN V2: A CRISPR/dCas9-Based Lateral Flow Assay for Rapid Detection of Theophylline**

Wenjun Jiang, Rashid Aman, Zahir Ali, and Magdy Mahfouz

*Laboratory for Genome Engineering and Synthetic Biology, Division of Biological Sciences, 4700 King Abdullah University of Science and Technology (KAUST), Thuwal 23955-6900, Saudi Arabia.*

†**Correspondence:** Magdy M. Mahfouz (magdy.mahfouz@kaust.edu.sa)

## Supplementary Tables

**Supplementary Table 1.** Oligonucleotide sequences for ligRNA template production.

| Group | Sequence (5′–3′) |
|---|---|
| T7-F | TTCTAATACGACTCACTATAGG |
| ligRNA1-1 | TTCTAATACGACTCACTATAGGGTACGCAGAAGGGAGCAGAGGGTTTCAGAG CTATGCTGGAAACAGCATAGCAAGTTGAAATAAGGGTG |
| ligRNA1-2 | AAAAAAGCACCGACTCGGTGCCGTCGCTGCCAAGGGCCTTTCGGCTGGTATC GGCGTATACGGGACACCCTTATTTCAACTTGC |
| ligRNA1-R | AAAAAAGCACCGACTCGGT |
| ligRNA2-1 | TTCTAATACGACTCACTATAGGGTACGCAGAAGGGAGCAGAGGGTTTTAGAG CTAGAAATAGCAAGTTAAAATAAGGCTAGTCCGTTATCAACTTGAA |
| ligRNA2-2 | CTAAAACCTGCCAAGGGCATCAAGACGATGCTGGTATGTTTTAGAGCTAGAA ATAAAAAGCACCGACTCGGTGCCACTTTTTCAAGTTGATAACGGACTAGC |
| ligRNA2-R | CTAAAACCTGCCAAGGGCATC |
| ligRNA3-1 | TTCTAATACGACTCACTATAGGGTACGCAGAAGGGAGCAGAGGGTTTCAGAG CTATGCTGGAAACAGCATAGCAAGTTGAAATAAGGCTAGTCCGTTATCAACT TGAAAAAGTGGCACCGAGTCGGTGCTTTTTCCTC |
| ligRNA3-2 | CACCGACTCCTGCCAAGGGCATCAAGACGATGCTGGTATCGAGTCGGTGCTT TTTGAGGAAAAAGCACCGACTCGG |
| ligRNA3-R | CACCGACTCCTGCCAAGGG |
| ligRNA4-1 | TTCTAATACGACTCACTATAGGGTACGCAGAAGGGAGCAGAGGGTTTCAGAG CTATGCTGGAAACAGCATAGCAAGTTGAAATAAGGCTAGTCCGTTATCAACT TGAAAAAGTGGCACCGAGTCGGTGCTTTTTCCTC |
| ligRNA4-2 | CACCGACTCGCTGCCAAGGGCATCAAGACGATGCTGGTATCCGAGTCGGTGC TTTTTGAGGAAAAAGCACCGACTCGGTGCCAC |
| ligRNA4-R | CACCGACTCGCTGCCAAGGG |

**Supplementary Table 2.** Oligonucleotide sequences for FAM-labeled amplicon production and *in vitro* Cas9 target cleavage.

| Group | Sequence (5′–3′) |
|---|---|
| Target-F-FAM | /56FAM/CAACTTCCTCAAGGAACAACATTGCCAAAA |
| Target-F-2 | ACCGAAGAGCTACCAGACG |
| Target-R | ATTCTAGCAGGAGAAGTTCCCCTACT |
| TheoPCR-template | ATTGGCACCCGCAATCCTGCTAACAATGCTGCAATCGTGCTACAACTTCCTCAAGGAACAACATTGCCAAAAGGCTTCTACGCAGAAGGGAGCAGAGGCGGCAGTCAAGCCTCTTCTCGTTCCTCATCACGTAGTCGCAACAGTTCAAGAAATTCAACTCCAGGCAGCAGTAGGGGAACTTCTCCTGCTAGAATGGCTGGCAATGGCGGTGATGCTGCTCTTGCTTTGCTGCTGCTTGACAGATTGAACCAGCTTGAGAGCAAAATGTCTGGTAAAGGCCAACAACAACAAGGCCAAACTGTCACTAAGAAATCTGCTGCTGAGGCTTCTAAGAAGCCTCGGCAAAAACGTACTGCCACTAAAGCATACAATGTAACACAAGCTTTCGGCAGACGTGGTCCAGAACAAACCCAAGGAAATTTTGGGGACCAGGAACTAATCAGACAAGGAACTGATTACAAACATTGGCCGCAAATTGCACAATTTGCCCCCAGCGCTTCAGCGTTCTTCGGAATGTCGCGCATTGGCATGGAAGTCACACCTTCGGGAACGTGGTTGACCTACACAGGTGCCATCAAATTGGATGACAAAGATCCAAATTTCAAAGATCAAGTCATTTTGCTGAATAAGCATATTGACGCATACAAAACATTCCCACCAACAGAGCC |

**Supplementary Table 3.** Sequences of ligRNAs used in this study.

| Group | Sequence (5′–3′) |
|---|---|
| ligRNA1 | UACGCAGAAGGGAGCAGAGGGUUUCAGAGCUAUGCUGGAAACAGCAUAGCAAGUUGAAAUAAGGGUGUCCCGUAUACGCCGAUACCAGCCGAAAGGCCCUUGGCAGCGACGGCACCGAGUCGGUGCUUUUUU |
| ligRNA2 | UACGCAGAAGGGAGCAGAGGGUUUUAGAGCUAGAAAUAGCAAGUUAAAAUAAGGCUAGUCCGUUAUCAACUUGAAAAAGUGGCACCGAGUCGGUGCUUUUUAUUUCUAGCUCUAAAACAUACCAGCAUCGUCUUGAUGCCCUUGGCAGGUUUUAG |
| ligRNA3 | UACGCAGAAGGGAGCAGAGGGUUUCAGAGCUAUGCUGGAAACAGCAUAGCAAGUUGAAAUAAGGCUAGUCCGUUAUCAACUUGAAAAAGUGGCACCGAGUCGGUGCUUUUUCCUCAAAAAGCACCGACUCGAUACCAGCAUCGUCUUGAUGCCCUUGGCAGGAGUCGGUG |
| ligRNA4 | UACGCAGAAGGGAGCAGAGGGUUUCAGAGCUAUGCUGGAAACAGCAUAGCAAGUUGAAAUAAGGCUAGUCCGUUAUCAACUUGAAAAAGUGGCACCGAGUCGGUGCUUUUUCCUCAAAAAGCACCGACUCGGAUACCAGCAUCGUCUUGAUGCCCUUGGCAGCGAGUCGGUG |
